# Ideal optical antimatter using passive lossy materials under complex frequency excitation

**DOI:** 10.1038/s41377-025-02137-w

**Published:** 2026-01-04

**Authors:** Olivia Y. Long, Peter B. Catrysse, Seunghoon Han, Shanhui Fan

**Affiliations:** 1 Department of Applied Physics, Stanford University, Stanford, CA 94305 USA; 2 Edward L. Ginzton Laboratory, Stanford University, Stanford, CA 94305 USA; 3 Samsung Advanced Institute of Technology, Samsung Electronics, Suwon-si, 16678 South Korea; 4 Semiconductor R&D Center, Samsung Electronics, Hwaseong-si, 18448 South Korea; 5 Department of Electrical Engineering, Stanford University, Stanford, CA 94305 USA

**Keywords:** Metamaterials, Transformation optics, Nanophotonics and plasmonics

## Abstract

The original concept of left-handed material has inspired the possibility of optical antimatter, where the effect of light propagation through a medium can be completely canceled by its complementary medium. Despite recent progress in the development of negative-index metamaterials, losses continue to be a significant barrier to realizing optical antimatter. In this work, we show that passive, lossy materials can be used to realize optical antimatter when illuminated by light at a complex frequency. We further establish that one can engineer arbitrary complex-valued permittivity and permeability in such materials. Strikingly, we show that materials with a positive index at real frequencies can act as negative-index materials under complex frequency excitation. Using our approach, we numerically demonstrate the optical antimatter functionality, as well as double focusing by an ideal perfect lens and superscattering. Our work demonstrates the power of temporally structured light in unlocking the promising opportunities of complementary media, which have until now been inhibited by material loss.

## Abstract (web summary)

Passive, lossy materials with positive index at real frequencies can be used to realize optical antimatter under complex frequency excitation. Our approach enables arbitrary complex $\epsilon ,\,\mu$ to be achieved.

## Graphical abstract

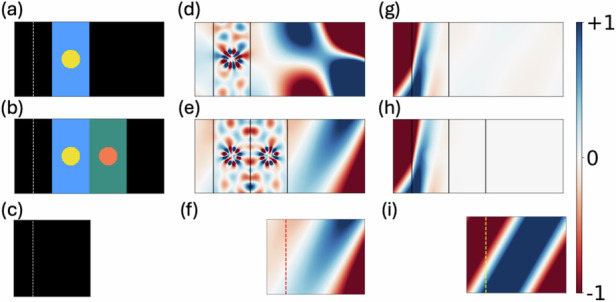

## Introduction

An intriguing effect enabled by the developments of metamaterials is the possibility of creating optical antimatter or complementary media. The electromagnetic scattering of a medium with an inhomogeneous distribution of permittivity $\epsilon$ and permeability $\mu$ can be completely eliminated, if one places a medium with complementary permittivity and permeability distribution $-\epsilon ,-\mu$ next to it^1^. The notion of optical antimatter is a generalization of the effect of perfect lensing with negative refraction^2,3^, and has further implications for transformation optics, ranging from superscattering^4–7^ to perfect invisibility devices^8–12^, which generally require spatially inhomogeneous materials. In theory, complementary media can give rise to a wealth of unique physical phenomena by effectively canceling the optical path traversed through a given region. Such exotic effects include cloaking^13–15^, illusion optics^16^, the construction of pseudo-Hermitian systems^17^, and subsurface imaging^18^. Thus, the realization of complementary media is of fundamental interest and would unlock new regimes of optical behaviors.

For a regular material with a positive permittivity and permeability, its complementary medium is a negative index medium with negative permittivity and permeability. However, due to the constraint of a positive energy density, negative permittivity or permeability necessarily implies that the material is dispersive^19^. By the Kramers–Kronig relations, then, such a material must also be lossy. In experiments, the presence of loss has severely limited the demonstration of such complementary media^20–27^.

In recent years, there have been emerging interests in considering the electromagnetic properties of materials at complex frequencies^28,29^. In particular, it has been theoretically proposed and experimentally demonstrated that the use of complex frequencies can significantly improve the resolution of a negative refraction lens where the material has intrinsic losses^30–33^. Many other effects associated with the use of complex frequency excitations, such as coherent virtual absorption^34^, virtual parity-time symmetry^35^, polariton propagation^36^, light stopping^37^, virtual critical coupling^38,39^, and transient non-Hermitian skin effect^40^ have also been considered. Building upon these works, here we show that a pair of complementary media can be constructed from only *lossy* materials with the use of complex frequency excitations. Remarkably, we show that such complementary media can be achieved using only *positive-index* dispersive, passive materials. In doing so, we also establish the possibility of engineering arbitrary complex-valued permittivity and permeability in such materials. We further demonstrate that our approach can be applied to realize optical antimatter, double refocusing, and superscattering. Our work opens an avenue to the experimental exploration of optical antimatter by overcoming the effects of loss that are intrinsic to these systems.

## Results

### Theory

We start by briefly reviewing the concept of complementary media, as originally proposed in Ref.^1^. For an isotropic medium with an electric permittivity $\epsilon$ and a magnetic permeability $\mu$, its complementary medium has an electric permittivity $\widetilde{\epsilon }$ and a magnetic permeability $\widetilde{\mu }$ that satisfies:

1 $$\begin{array}{c}\widetilde{\epsilon }=-\epsilon ,\widetilde{\mu }=-\mu \end{array}$$

Moreover, Ref.^1^ envisioned an ideal scenario where the two complementary media are both lossless. Here in this section, we show that such an ideal complementary pair can be realized at a single complex frequency using lossy, passive materials.

#### Lossless propagation in lossy, passive materials at complex frequency

We first discuss the condition for lossless propagation of light at a complex frequency. This requires the wavevector $k$ to be purely real. Using the ${e}^{-i\omega t}$ time convention, Maxwell’s equations state

2 $$k\times E=\omega \mu (\omega )H$$

3 $$k\times H=-\omega \epsilon (\omega )E$$

where we have explicitly included the frequency dependence of $\epsilon$ and $\mu$. When $\omega {\mathbb{\in }}{\mathbb{C}}$, the following conditions

4 $$\omega \epsilon \left(\omega \right){\mathbb{\in }}{\mathbb{R}}$$

5 $$\omega \mu \left(\omega \right){\mathbb{\in }}{\mathbb{R}}$$

are sufficient in order to have $k{\mathbb{\in }}{\mathbb{R}}$. Importantly, at a complex frequency, to achieve lossless propagation of light, the permittivity and permeability must also be complex.

We now proceed to show that a complementary media pair with lossless propagation in both media can be achieved using lossy, passive materials excited at a complex frequency. For this purpose, we consider the Lorentz-Drude model, which is both a fundamental model for a dispersive material and a common model used in the design of metamaterials^41^:

6 $$\epsilon \left(\omega \right)=1+\frac{{\omega }_{p}^{2}}{{\omega }_{0}^{2}-{\omega }^{2}-i\omega \gamma }$$

In this model, ${\omega }_{0}$ is the frequency of the material resonance, and hence:

7 $${\omega }_{0}^{2} > 0$$

The plasma frequency is ${\omega }_{p}$, and passivity requires that^42^

8 $${\omega }_{p}^{2} > 0$$

while causality requires the damping rate $\gamma$ to satisfy^43^

9 $$\gamma > 0$$

We note that the Lorentz–Drude model is applicable to the entire complex frequency plane and is, in fact, the most general model for the response functions of permittivity and permeability. Here, we employ the single-pole Lorentz–Drude model, which is valid provided that the operating frequency (which can be complex) is in the vicinity of the pole. When the operating frequency has a small imaginary part, one can use the same poles that describe the real-frequency response^44^. For a general complex frequency, the pole assumed may not be the same pole used to describe the real-frequency response.

Our argument is based on the following theorem.

**Theorem 1**. *Given a complex frequency* $\omega ={\omega }^{{\prime} }+i{\omega }^{{\prime} {\prime} }$ *with* ${\omega }^{{\prime} {\prime} } < 0$*, and any complex value* $C+{iD}$ *where* $C,D{\mathbb{\in }}{\mathbb{R}}$*, there exist parameters* ${\omega }_{0}^{2},{\omega }_{p}^{2},\gamma$ *satisfying Eqs.* *(**7**)–(**9**)* *such that* $\epsilon \left(\omega \right)=C+{iD}$.

We now prove this theorem. Equating the Lorentz–Drude expression in Eq. (6) to $C+{iD}$ and matching the real and imaginary parts yields a system of equations described by

10 $$M{\bf{x}}={\bf{b}}$$

where:

11 $$M=\left[\begin{array}{ccc}C-1 & 1 & {\omega }^{{\prime\prime} }\left(C-1\right)-{\omega }^{{\prime} }D\\ D & 0 & {\omega }^{{\prime} }\left(C-1\right)+{\omega }^{{\prime\prime} }D\end{array}\right]$$

12 $${\bf{x}}=\left[\begin{array}{c}{\omega }_{0}^{2}\\ {\omega }_{p}^{2}\\ \gamma \end{array}\right]$$

13 $${\bf{b}}=\left[\begin{array}{c}\left(C-1\right)\left({\omega^{\prime} }^{2}-{\omega^{\prime\prime}}^{2}\right)+2{\omega }^{{\prime} }{\omega }^{{\prime\prime} }D\\ D\left({\omega^{\prime} }^{2}-{\omega^{\prime\prime}}^{2}\right)-2{\omega }^{{\prime} }{\omega }^{{\prime\prime} }\left(C-1\right)\end{array}\right]$$

We thus wish to show that there always exists a solution ${\bf{x}}$ with all components of ${\bf{x}}$ being positive for arbitrary $C,D{\mathbb{\in }}{\mathbb{R}}$.

We first identify a particular solution:

14 $${{\bf{x}}}_{p}=\left[\begin{array}{c}{\omega^{\prime} }^{2}+{\omega^{\prime\prime} }^{2}\\ 0\\ {-2\omega }^{{\prime\prime} }\end{array}\right]$$

Since we have set ${\omega }^{{\prime\prime} } < 0$, the nonzero components of ${{\bf{x}}}_{p}$, i.e., ${\omega }^{{\prime} 2}+{\omega }^{{\prime} {\prime} 2},-2{\omega }^{{\prime} {\prime} } > 0$.

Since the system of equations in Eq. (10) is underdetermined, the complete solution ${\bf{x}}$ is:

15 $${\bf{x}}={{\bf{x}}}_{p}+\alpha {{\bf{x}}}_{n}$$

where $\alpha$ is a free variable and

16 $${{\bf{x}}}_{n}={{\bf{r}}}_{2}\times {{\bf{r}}}_{1}=\left[\begin{array}{c}{\omega }^{{\prime} }\left(1-C\right)-{\omega }^{{\prime} {\prime} }D\\ {\omega }^{{\prime} }\left({D}^{2}+{\left(C-1\right)}^{2}\right)\\ D\end{array}\right]$$

where ${{\bf{r}}}_{1}$ and ${{\bf{r}}}_{2}$ are the first and the second rows of $M$, respectively. Equation (15) can be derived by noting that $M{{\bf{x}}}_{n}={\bf{0}}$. We now perform a component-wise analysis. Let ${x}_{k}$ denote the *k*th component of ${\bf{x}}$. From Eq. (15), each component can thus be written as:

17 $${x}_{k}\left(\alpha \right)={\left({x}_{p}\right)}_{k}+\alpha {\left({x}_{n}\right)}_{k}$$

Without loss of generality, we can assume ${\omega }^{{\prime} } > 0$. In this case, ${x}_{2}\left(\alpha \right) > 0$ for any choice of $\alpha > 0$. On the other hand, since ${\left({x}_{p}\right)}_{1}$ and ${\left({x}_{p}\right)}_{3}$ are both positive, with a sufficiently small $\alpha > 0$, by continuity, both ${x}_{1}\left(\alpha \right)$ and ${x}_{3}\left(\alpha \right)$ remain positive. Therefore, there exists a solution ${\bf{x}}$ with all its components being positive. This concludes our proof of the theorem. With a bit of further algebra, the procedure above, in fact, can be used to determine the range of $\alpha$ in which the components of ${\bf{x}}$ are all positive. Detailed derivations can be found in the Supplementary Materials. The theorem above can likewise be applied to $\mu \left(\omega \right)$, which can also be described by a similar Lorentz–Drude model^41^.

In metamaterials, the parameters ${\omega }_{p}^{2}$, ${\omega }_{0}^{2}$, and $\gamma$ can all be controlled: ${\omega }_{p}^{2}$ is related to the density of meta-atoms, ${\omega }_{0}^{2}$ is controlled by the geometry of an individual meta-atom, and $\gamma$ is related to the choice of constituent materials. Our theorem thus indicates substantial new flexibility in achieving arbitrary electromagnetic response, using lossy passive metamaterials, by operating at complex frequencies.

We apply the theorem above to the design of optical antimatter. At a given complex frequency $\omega$ with ${\omega }^{{\prime} {\prime} } < 0$, we can choose two arbitrary real positive numbers ${q}_{\epsilon }$ and ${q}_{\mu }$. From our theorem, there exists a pair of lossy, passive Lorentz-Drude materials as described by $\epsilon \left(\omega \right)$, $\mu \left(\omega \right)$ and $\widetilde{\epsilon }\left(\omega \right)$, $\widetilde{\mu }\left(\omega \right)$, respectively, such that

18 $$\widetilde{\epsilon }\left(\omega \right)=-\epsilon \left(\omega \right)=\frac{{q}_{\epsilon }}{\omega }$$

19 $$\widetilde{\mu }\left(\omega \right)=-\mu \left(\omega \right)=\frac{{q}_{\mu }}{\omega }$$

These materials form a complementary pair that both support lossless propagation.

#### Achieving negative index behavior in positive index media at complex frequency

As can be seen in Eq. (1), complementary media are closely related to negative index media. As a side note, we observe that a medium that has a positive index at a real frequency may possess a negative index at a complex frequency with the same real component. To illustrate this point, we consider a medium with a permittivity $\epsilon \left(\omega \right)$ and a permeability ${\mu }_{0}$.

Without loss of generality, we consider TM-polarized waves propagating in the $z$-direction:

20 $${\bf{E}}\left(z\right)={e}^{{ikz}}\left({E}_{x},0,0\right)$$

21 $${\bf{H}}\left(z\right)={e}^{{ikz}}\left(0,{H}_{y},0\right)$$

Expressing the refractive index $n$ in terms of its real and imaginary components: $n={n}^{{\prime} }+i{n}^{{\prime} {\prime} }$. The wavevector $k$ can then be written as:

22 $$\begin{array}{c}k=\frac{\omega n}{c}=\frac{\left({\omega }^{{\prime} }+{i\omega }^{{\prime\prime} }\right)\left({n}^{{\prime} }+{{in}}^{{\prime\prime} }\right)}{c}\\ =\frac{\left({\omega }^{{\prime} }{n}^{{\prime} }-{\omega }^{{\prime\prime} }{n}^{{\prime\prime} }\right)+i\left({\omega }^{{\prime} }{n}^{{\prime\prime} }+{\omega }^{{\prime\prime} }{n}^{{\prime} }\right)}{c}\end{array}$$

Thus, the direction of the phase velocity is controlled by the sign of $\text{Re}\left[k\right]={\omega }^{{\prime} }{n}^{{\prime} }-{\omega }^{{\prime} {\prime} }{n}^{{\prime} {\prime} }$. On the other hand, the direction of the power flow is determined by the sign of the $z$-component of the time-averaged Poynting vector $\left\langle {\bf{S}}\right\rangle =\frac{1}{2}\text{Re}\left[{\bf{E}}\times {{\bf{H}}}^{* }\right]$:

23 $$\left\langle {S}_{z}\right\rangle =\frac{{|{E}_{x}|}^{2}}{2}\sqrt{\frac{{\epsilon }_{0}}{{\mu }_{0}}}{n}^{{\prime} }{e}^{-\,\frac{2\left({\omega }^{{\prime} }{n}^{{\prime} {\prime} }+{\omega }^{{\prime} {\prime} }{n}^{{\prime} }\right)}{c}}$$

Here, the sign of ${n}^{{\prime} }$ determines the direction of $\left\langle {S}_{z}\right\rangle$. When $\omega$ is real, i.e., ${\omega }^{{\prime} {\prime} }=0$, the phase and the group velocity have the same sign, as expected since this material has a magnetic permeability of ${\mu }_{0}$^45^. However, for a complex $\omega$, it becomes possible to have ${\omega }^{{\prime} {\prime} }{n}^{{\prime} {\prime} } > {\omega }^{{\prime} }{n}^{{\prime} }$, while keeping the sign of ${n}^{{\prime} }$ to be positive. Thus, negative index behavior can be achieved at the complex $\omega$.

Having established the theoretical basis for complementary media with lossless propagation using only passive, lossy materials at a complex frequency, we next illustrate the optical antimatter functionality with numerical simulations. Although there has been experimental work on optical antimatter for air, the impedance mismatch between air and the structure prevents the full recovery of all transverse wave components^46^. Moreover, there have not been any works to date on optical antimatter for materials that are inhomogeneous in the transverse direction. Such inhomogeneity would pave the way to a broader class of applications beyond lensing, including superscattering and cloaking.

### Numerical demonstrations

#### Optical antimatter

In our numerical demonstrations, we employ a temporally decaying wave with complex frequency $\omega ={\omega }_{c}=\left(1.256\times {10}^{15}-4\times {10}^{14}i\right)$ rad/s. Note that $\text{Im}\left[{\omega }_{c}\right] < 0$, consistent with our theory. The carrier frequency ${\omega }_{r}=\text{Re}\left[{\omega }_{c}\right]$ corresponds to light with a free-space wavelength of 1.5 μm. We use two pairs of complementary media described by passive, lossy Lorentz-Drude models. The parameters ${\omega }_{p}^{2},{\omega }_{0}^{2},\gamma$, as well as the values of $\epsilon$ and $\mu$ for these media at frequencies $\omega ={\omega }_{c}$ and $\omega ={\omega }_{r}$, are provided in Table 1.

**Table 1:** Material parameters of Media 1–4 used in numerical demonstrations

Medium	Parameter	$\omega ={\omega }_{c}$	$\omega ={\omega }_{r}$	${\omega }_{p}({10}^{15}{rad}/s)$	${\omega }_{0}({10}^{15}{rad}/s)$	$\gamma ({10}^{15}{rad}/s)$
$1$	${\varepsilon }_{1}$	4 + 1.274*i*	$3.289+1.292i$	$3.873$	$2.559$	$2.233$
	${\mu }_{1}$	6 + 1.911*i*	$1.776+1.513i$	$1.483$	$1.472$	$0.917$
$2$	${\epsilon }_{2}$	−4 – 1.274*i*	$0.756+1.570i$	$1.225$	$1.197$	$0.743$
	${\mu }_{2}$	−6 – 1.911*i*	$0.890+1.627i$	$1.245$	$1.230$	$0.755$
$3$	${\epsilon }_{3}$	12 + 3.822*i*	$2.812+3.399i$	$2.236$	$1.479$	$0.912$
	${\mu }_{3}$	15 + 4.778*i*	$1.474+1.682i$	$1.378$	$1.368$	$0.833$
$4$	${\epsilon }_{4}$	−12 – 3.822*i*	$1.051+1.752i$	$1.304$	$1.267$	$0.772$
	${\mu }_{4}$	−15 – 4.778*i*	$1.097+1.684i$	$1.284$	$1.278$	$0.777$

${\omega }_{c}=\left(1.256\times {10}^{15}-4\times {10}^{14}i\right)$ rad/s and ${\omega }_{r}=\text{Re}\left[{\omega }_{c}\right]=1.256\times {10}^{15}$ rad/s

We note that the resonant response in the magnetic permeability, as described by the Lorentz–Drude model, has been observed in optical frequencies using meta-atoms that support magnetic dipole resonances^47–49^. Similar resonant response in the electric permittivity can be achieved with the use of systems exhibiting resonant electric dipole response, such as dielectric meta-atoms^50,51^. These systems have not been previously used to demonstrate optical antimatter due to the substantial loss. The use of complex frequencies, as we propose here, overcomes the challenges associated with the loss.

The first complementary pair is comprised of Medium 1 and 2. At $\omega ={\omega }_{c}$, ${\epsilon }_{2}=-{\epsilon }_{1}$ and ${\mu }_{2}=-{\mu }_{1}$. The second pair is comprised of Medium 3 and 4, where similarly at $\omega ={\omega }_{c}$, ${\epsilon }_{4}=-{\epsilon }_{3}$ and ${\mu }_{4}=-{\mu }_{3}$. At $\omega ={\omega }_{c}$, these materials satisfy Eqs. (4), (5) such that the wavevector is purely real and there is lossless propagation of light in each media.

**Fig. 1:**
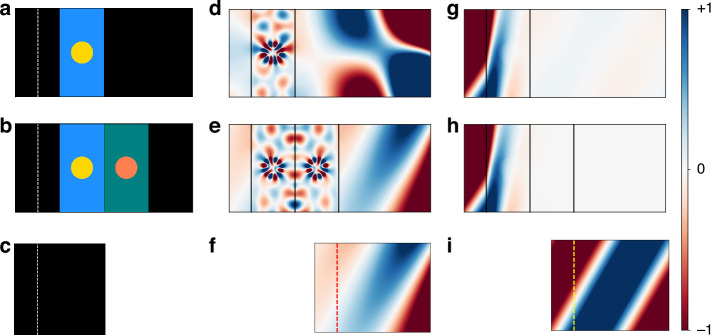
Optical antimatter at complex frequency. In panels (**a**)–(**c**), the colors blue, teal, yellow, and orange correspond to Media 1, 2, 3, and 4, as described in Table 1, respectively. Black denotes vacuum. **a** Schematic of the initial structure with a circular scatterer of radius 150 nm embedded in a slab of thickness 0.6 μm. The dashed white line indicates the location of the TM-polarized plane wave source. **b** Schematic of structure placed adjacent to its complementary counterpart. **c** Free space configuration with plane wave source. **d**–**f** TM-polarized Re[${H}_{z}$] field patterns at the complex frequency $\omega ={\omega }_{c}$ for the configurations in (**a**)–(**c**), respectively. **g**–**i** TM-polarized Re[${H}_{z}$] field patterns at the real frequency $\omega ={\omega }_{r}$ for the configurations in (**a**)–(**c**), respectively. In **f**, **i**, the red and yellow dashed lines mark the effective location of the optically canceled complementary media pair, respectively. For each $\omega$, field plots are normalized to the same maximum and minimum values. All field plots were generated using FDFD^60^, with Bloch boundary conditions in the transverse ($y$) direction and PML boundary conditions in the propagation ($x$) direction. Plots show the 0th diffraction order in free space

In Fig. 1, we showcase the functionality of optical antimatter. Figure 1a depicts the first structure, where a circular scatterer comprised of Medium 3 is embedded in a slab of Medium 1 that is surrounded by free space. The slab has a thickness of 0.6 μm, and the scatterer has a radius of $150$ nm. The structure is excited by a TM-polarized plane wave at $\omega ={\omega }_{c}$, launched from a line source in free space [dashed white line in Fig. 1a] and incident at an angle of 30° with respect to the horizontal direction. For reference, the same source and the corresponding field pattern in free space are shown in Fig. 1c, f, respectively. Note that the fields in free space are spatially growing along the propagation direction due to the complex frequency excitation. In other words, the wavevector in the background is complex and exhibits effective gain in the $+x$ direction, as depicted by the increasing amplitude of the fields propagating in the free space regions of Fig. 1d–f.

When the incident plane wave is transmitted through the structure in Fig. 1a, the field pattern that emerges on both sides of the slab, as shown in Fig. 1d, is markedly different from that of the plane wave excitation, indicating the presence of strong reflection and scattering.

To demonstrate optical antimatter, we now place the complementary structure adjacent to the first structure, as depicted schematically in Fig. 1b. Remarkably, in Fig. 1e, we see that the field patterns outside the combined structure on the left and right sides match precisely those in free space on the left and right sides of the red line in Fig. 1f, respectively. The presence of the complementary structure thus cancels all optical effects of the first structure, rendering the combined structure invisible to the incident light. We also note that the field pattern inside the combined structure has a mirror symmetry with respect to the interface between the first structure and its complement, even though the field is incident from the left. Such a mirror symmetry is expected for such complementary media, as proven in Ref.^1^.

We next excite the structures with a TM-polarized plane wave at the corresponding real frequency $\omega ={\omega }_{r}$, with the field pattern in free space shown in Fig. 1i. As expected, the excitation field propagates in free space without growth or decay along the propagation direction. At $\omega ={\omega }_{r}$, the relative permittivity and permeability values for all four media are shown in Table 1. Notably, all refractive indices are positive. Moreover, all media are lossy, as expected for such passive, lossy Lorentz-Drude models^43^. The resulting field patterns for the first structure and for the combined structure are shown in Fig. 1g, h, respectively. We see that in both cases, the field patterns outside the structure deviate significantly from those of free space. Thus, at this real frequency, the combined structure no longer exhibits the effects of optical antimatter. For additional optical antimatter demonstrations using different geometries, see Supplementary Materials.

#### Ideal perfect lens

**Fig. 2:**
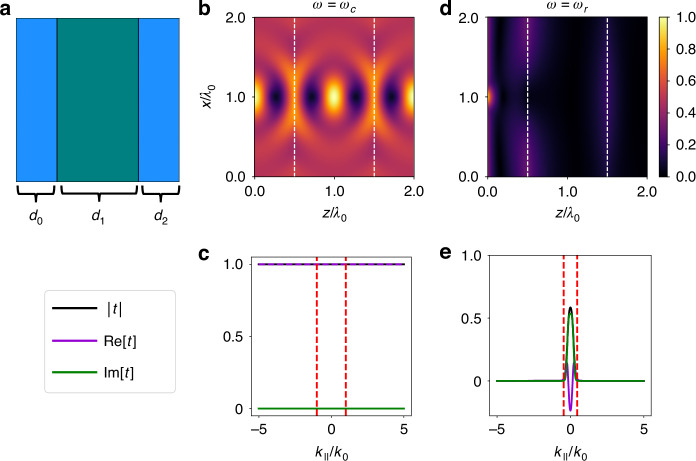
The double focusing effect of an ideal perfect lens is enabled by optical antimatter under complex frequency excitation. **a** Schematic of three layers comprised of Medium 1, Medium 2, and Medium 1 depicted using the color scheme of Fig. 1. Layer thicknesses from left to right: ${d}_{0}=0.5{\lambda }_{0},\,{d}_{1}={\lambda }_{0},\,{d}_{2}=0.5{\lambda }_{0}$ where ${\lambda }_{0}$ is the wavelength in Medium 1 at $\omega ={\omega }_{c}$. **b** Magnitude of Poynting vector for a Gaussian point source originating in the leftmost layer at complex frequency $\omega ={\omega }_{c}$ and at **d** real frequency $\omega ={\omega }_{r}$ normalized to their respective maximum values. Dashed white lines indicate interfaces between Medium 1 and Medium 2. For clarity, only propagating waves are plotted in the complex frequency case. **c** Transmission coefficient through setup is shown in panel **a** at $\omega ={\omega }_{c}$ and at **e** $\omega ={\omega }_{r}$. In both plots, ${k}_{0}$ refers to the wavevector defined in Medium 1 at $\omega ={\omega }_{c}$. Red dashed lines mark the boundary between evanescent and propagating waves. All plots shown are for TM polarization

One application of complementary media is for the construction of a perfect lens^2^. As a second illustration, we show that an ideal perfect lens can be achieved using passive, lossy Lorentz–Drude materials excited at a complex frequency. We consider three planar slabs of material comprised of Medium 1, Medium 2, and Medium 1, respectively, as depicted in Fig. 2a. From left to right, the layers have thicknesses ${d}_{0}=0.5{\lambda }_{0},{d}_{1}={\lambda }_{0},{d}_{2}=0.5{\lambda }_{0}$, where ${\lambda }_{0}\approx 277.98$ nm, the wavelength in both media at $\omega ={\omega }_{c}$. The associated wavevector ${k}_{0}=2{\rm{\pi }}/{{\rm{\lambda }}}_{0}$ is purely real. The slab thicknesses were chosen such that the source could be refocused twice: once inside the central slab and once at the end of the rightmost slab. This was a design choice to showcase the double-focusing phenomenon of the perfect lens. However, the slab thicknesses can be tuned based on the desired locations of the foci and do not affect the lens performance.

In this setup, a line source with a Gaussian spatial distribution with standard deviation $0.1{\lambda }_{0}$ is placed in the leftmost layer of Medium 1 at $\left(x,z\right)=\left(1,0\right){\lambda }_{0}$, as shown in Fig. 2b. The plot shows the magnitude of the Poynting vector when the source has complex frequency $\omega ={\omega }_{c}$. We see that the field is refocused at the midpoint of layer 2 and again at the rightmost point of layer 3, achieving the double focusing effect of an ideal perfect lens^2^. For visual clarity, the plot shows only propagating waves due to the exponential growth of the evanescent waves. The transmission coefficient through the three-layer configuration plotted as a function of the parallel wavevector ${k}_{\parallel }$ is shown in Fig. 2c. The red dashed lines mark the boundaries between propagating and evanescent waves, which occur at ${k}_{\parallel }=\pm {k}_{0}$. We see that at complex frequency, the transmission is unity for both propagating and evanescent waves. Moreover, the phase remains unaffected, since $\text{Im}\left[t\right]=0$. As a comparison, at the real frequency $\omega ={\omega }_{r}$, no double focusing behavior is observed (Fig. 2d, e), as expected since the two media have positive indices at $\omega ={\omega }_{r}$.

Our results here indicate that the ideal perfect lens, which performs both refocusing of propagating waves and recovery of evanescent waves, can be achieved using passive lossy media operating at complex frequencies. We note that the results here differ from those in Ref.^31^, which focused on the recovery of evanescent field components, corresponding to the so-called “poor-man’s superlens,” as defined by Pendry^2^. In general, there is a high sensitivity of the evanescent waves to impedance mismatches in the lens system^52^. Here, we emphasize that the key advantage in our approach is that perfect effective impedance matching can be achieved, yielding unity transmission for all propagating and evanescent waves. Additional plots and analyses of lensing using imperfect complementary media and finite bandwidth sources can be found in the Supplementary Materials.

#### Superscattering

As a final example, we demonstrate the phenomenon of superscattering that is enabled by optical antimatter at complex frequency. Theoretical proposals for superscatterers hinge on the use of ideal negative-index materials^4,53^ or multiple overlapping resonances in plasmonic-dielectric-plasmonic layered structures^6,54^. In both cases, the experimental realization of superscatterers, especially at optical frequencies, is hindered by the loss present in the required materials. Here, we show that the effects of superscattering can be achieved with passive, lossy materials at a complex frequency.

**Fig. 3:**
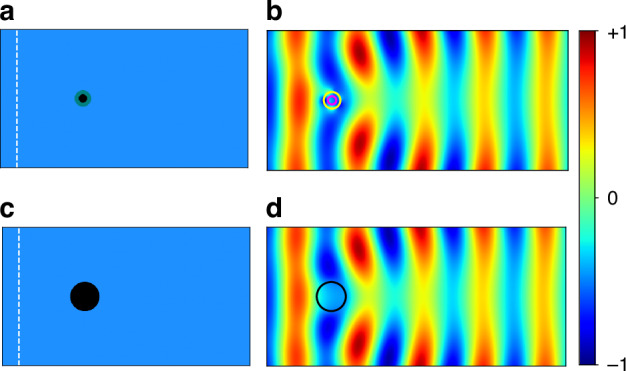
Superscattering using optical antimatter at complex frequency. **a** Schematic of air scatterer with radius $r=18.75$ nm embedded in Medium 1 and surrounded by an annulus of complementary Medium 2 with thickness $18.75$ nm. **b** Field distribution of Re[${H}_{z}$] in panel **a** under plane wave excitation at complex frequency from left. The yellow circle indicates the boundary between the annulus and the surrounding medium. The magenta circle indicates the boundary of the inner air scatterer. **c** Schematic of air scatterer with radius $R=66.6$ nm embedded in Medium 1. **d** Field distribution of Re[${H}_{z}$] in panel **c** under plane wave excitation at complex frequency from left. The black circle indicates the boundary of the air scatterer

In Fig. 3a, we consider a cylinder of air surrounded by an annulus of Medium 2, embedded in Medium 1. The radius of the air cylinder and the thickness of the annulus are both $18.75$ nm, giving an overall radius of $r=37.5$ nm. The outer radius was chosen to be twice that of the inner radius for direct comparison with the conception of the superscatterer proposed in Ref.^4^. The white dashed line in Fig. 3a denotes the location of the TM-polarized plane wave source. Since Media 1 and 2 form a complementary pair, the annulus optically cancels a portion of the embedding medium when excited at the complex frequency $\omega ={\omega }_{c}$. The resulting field distribution is shown in Fig. 3b. Due to the optical antimatter effects of the annulus, the field distribution appears as if scattering off a larger object. We validate this by comparing it to the scattering of an air cylinder with radius $R=66.6$ nm, as depicted in Fig. 3c. The field distributions of the two scatterers are almost identical, as can be seen in Fig. 3b, d. The two scatterers have the same scattering cross section ${\sigma }_{\text{scat}}=0.2767\mu {\rm{m}}$. On the other hand, the structure in Fig. 3c has a radius that is nearly twice that of the outer radius of the annulus in Fig. 3a. The results here illustrate the ability to use passive, lossy media to achieve a superscattering effect by operating at a complex frequency.

## Discussion

In summary, we have demonstrated the concept of optical antimatter using only *passive, lossy* materials at complex frequencies. We further established that one can engineer arbitrary complex-valued permittivity and permeability in such materials. We find that *positive-index* Lorentz-Drude materials can be harnessed to achieve effective *negative-index* materials when excited at complex frequency. Furthermore, we have shown that such a concept can be applied to achieve ideal negative index lensing and superscattering, which have both been difficult to realize due to losses, especially at optical frequencies. The realization of such complementary media requires the control of the positions of the poles in both permittivity and permeability, as well as the creation of complex frequency excitations. In the development of metamaterials, there have been extensive efforts seeking to control the positions of these poles^55–57^. Here, we no longer require poles that are nearly lossless, which should simplify the design of structures that exhibit the required poles. Complex frequency excitations can be realized experimentally using high-speed electro-optic modulators at near-infrared and visible wavelengths^28^ or using a multifrequency synthesis approach that reconstructs the complex frequency response from measurements at real frequencies^30,36^.

In this work, we have highlighted the interplay between complex frequency and complex material parameters to achieve lossless propagation within the media and effective refractive index manipulation. We emphasize that this approach is fundamentally different from that of real frequencies, since complex frequencies can unlock access to *all transverse wavevectors*, including evanescent waves, using *only passive materials*. This is impossible at real frequencies, since there will always be inherent material loss, which severely diminishes the transmission of evanescent waves^52^. Thus, the full capabilities of complementary media cannot be realized at real frequencies. Moreover, we have shown in this work that *negative-index materials are not needed* to realize complementary media at complex frequencies, since materials with *positive-index* can act as effective negative-index materials when operated at the appropriate complex frequency. Future directions include exploring other possibilities in transformation optics, such as invisibility cloaking^58^, as well as non-Hermitian phenomena such as exceptional points and perfect absorbers^59^. Our work points to avenues for using temporally structured light to realize exotic optical behaviors.

## Materials and methods

The electromagnetic field demonstrations of optical antimatter with complex frequency (Figs. 1 and 3) were computed using an open-source finite difference frequency domain (FDFD) code^60^. In Fig. 1, Bloch boundary conditions were applied to the transverse direction to impose the appropriate phase shift for the excitation of the off-normal incident light. Along the propagation direction, perfectly matched layer (PML) boundary conditions were used to absorb outgoing waves. The existing code was modified to accommodate double negative media ($\epsilon ,\mu < 0$) and to account for the complex frequency in the PML media. In Fig. 3, PML boundary conditions were applied along all directions to absorb the scattered waves. Scattering cross sections for the superscatterer and air cylinder in Fig. 3 were calculated using the total-field/scattered-field (TFSF) framework implemented within the FDFD simulations^61^. The TFSF code was adapted to accommodate background media with arbitrary permittivity and permeability. Further calculation details can be found in the Supplementary Materials. Field plots in Fig. 2 were computed using a scattering-matrix code specifically written to ensure numerical stability at complex frequency excitations^62^.

## Supplementary information

Supplemental Material

## Data Availability

Data underlying the results presented in this paper are not publicly available at this time but may be obtained from the authors upon reasonable request.

## References

[1] Pendry, J. B. & Ramakrishna, S. A. Focusing light using negative refraction. *J. Phys.* **15**, 6345–6364 (2003).

[2] Pendry, J. B. Negative refraction makes a perfect lens. *Phys. Rev. Lett.* **85**, 3966–3969 (2000). 11041972 10.1103/PhysRevLett.85.3966

[3] Veselago, V. G. The electrodynamics of substances with simultaneously negative values of *ϵ* and µ. *Sov. Phys. Uspekhi* **10**, 509–514 (1968).

[4] Wee, W. H. & Pendry, J. B. Shrinking optical devices. *N. J. Phys.* **11**, 073033 (2009).

[5] Mirzaei, A. et al. Cloaking and enhanced scattering of core-shell plasmonic nanowires. *Opt. Express* **21**, 10454–10459 (2013). 23669901 10.1364/OE.21.010454

[6] Ruan, Z. C. & Fan, S. H. Superscattering of light from subwavelength nanostructures. *Phys. Rev. Lett.* **105**, 013901 (2010). 20867445 10.1103/PhysRevLett.105.013901

[7] Verslegers, L. et al. From electromagnetically induced transparency to superscattering with a single structure: a coupled-mode theory for doubly resonant structures. *Phys. Rev. Lett.* **108**, 083902 (2012). 22463532 10.1103/PhysRevLett.108.083902

[8] Leonhardt, U. & Philbin, T. G. General relativity in electrical engineering. *N. J. Phys.* **8**, 247 (2006).

[9] Pendry, J. B., Schurig, D. & Smith, D. R. Controlling electromagnetic fields. *Science* **312**, 1780–1782 (2006). 16728597 10.1126/science.1125907

[10] Schurig, D. et al. Metamaterial electromagnetic cloak at microwave frequencies. *Science* **314**, 977–980 (2006). 17053110 10.1126/science.1133628

[11] Cai, W. S. et al. Optical cloaking with metamaterials. *Nat. Photonics* **1**, 224–227 (2007).

[12] Valentine, J. et al. An optical cloak made of dielectrics. *Nat. Mater.* **8**, 568–571 (2009). 19404237 10.1038/nmat2461

[13] Lai, Y. et al. Complementary media invisibility cloak that cloaks objects at a distance outside the cloaking shell. *Phys. Rev. Lett.* **102**, 093901 (2009). 19392518 10.1103/PhysRevLett.102.093901

[14] Zheng, B. et al. Concealing arbitrary objects remotely with multi-folded transformation optics. *Light* **5**, e16177 (2016). 10.1038/lsa.2016.177 PMC6059891 30167134

[15] Milton, G. W. & Nicorovici, N. A. P. On the cloaking effects associated with anomalous localized resonance. *Proc. R. Soc. A* **462**, 3027–3059 (2006).

[16] Lai, Y. et al. Illusion optics: the optical transformation of an object into another object. *Phys. Rev. Lett.* **102**, 253902 (2009). 19659076 10.1103/PhysRevLett.102.253902

[17] Luo, L. Y. et al. Pseudo-Hermitian systems constructed by transformation optics with robustly balanced loss and gain. *Adv. Photonics Res.* **2**, 2000081 (2021).

[18] Kobayashi, K. Complementary media of electrons. *J. Phys.* **18**, 3703–3720 (2006).

[19] Smith, D. R. & Kroll, N. Negative refractive index in left-handed materials. *Phys. Rev. Lett.* **85**, 2933–2936 (2000). 11005971 10.1103/PhysRevLett.85.2933

[20] Podolskiy, V. A. & Narimanov, E. E. Near-sighted superlens. *Opt. Lett.* **30**, 75–77 (2005). 15648643 10.1364/ol.30.000075

[21] Shelby, R. A., Smith, D. R. & Schultz, S. Experimental verification of a negative index of refraction. *Science* **292**, 77–79 (2001). 11292865 10.1126/science.1058847

[22] Grigorenko, A. N. et al. Nanofabricated media with negative permeability at visible frequencies. *Nature* **438**, 335–338 (2005). 16292306 10.1038/nature04242

[23] Shalaev, V. M. et al. Negative index of refraction in optical metamaterials. *Opt. Lett.* **30**, 3356–3358 (2005). 16389830 10.1364/ol.30.003356

[24] Liu, N. et al. Three-dimensional photonic metamaterials at optical frequencies. *Nat. Mater.* **7**, 31–37 (2008). 18059275 10.1038/nmat2072

[25] Soukoulis, C. M., Linden, S. & Wegener, M. Negative refractive index at optical wavelengths. *Science* **315**, 47–49 (2007). 17204630 10.1126/science.1136481

[26] Zheludev, N. I. The road ahead for metamaterials. *Science* **328**, 582–583 (2010). 20431006 10.1126/science.1186756

[27] Smith, D. R. et al. Limitations on subdiffraction imaging with a negative refractive index slab. *Appl. Phys. Lett.* **82**, 1506–1508 (2003).

[28] Kim, S., Krasnok, A. & Alù, A. Complex-frequency excitations in photonics and wave physics. *Science* **387**, eado4128 (2025). 40146845 10.1126/science.ado4128

[29] Lalanne, P. et al. Light interaction with photonic and plasmonic resonances. *Laser Photonics Rev.* **12**, 1700113 (2018).

[30] Guan, F. X. et al. Overcoming losses in superlenses with synthetic waves of complex frequency. *Science* **381**, 766–771 (2023). 37590345 10.1126/science.adi1267

[31] Kim, S. et al. Loss compensation and superresolution in metamaterials with excitations at complex frequencies. *Phys. Rev. X* **13**, 041024 (2023).

[32] Archambault, A., Besbes, M. & Greffet, J. J. Superlens in the time domain. *Phys. Rev. Lett.* **109**, 097405 (2012). 23002884 10.1103/PhysRevLett.109.097405

[33] Dubois, M. et al. Time-driven superoscillations with negative refraction. *Phys. Rev. Lett.* **114**, 013902 (2015). 25615470 10.1103/PhysRevLett.114.013902

[34] Baranov, D. G., Krasnok, A. & Alù, A. Coherent virtual absorption based on complex zero excitation for ideal light capturing. *Optica* **4**, 1457–1461 (2017).

[35] Li, H. N. et al. Virtual parity-time symmetry. *Phys. Rev. Lett.* **124**, 193901 (2020). 32469571 10.1103/PhysRevLett.124.193901

[36] Guan, F. X. et al. Compensating losses in polariton propagation with synthesized complex frequency excitation. *Nat. Mater.* **23**, 506–511 (2024). 38191633 10.1038/s41563-023-01787-8

[37] Tsakmakidis, K. L. et al. Completely stopped and dispersionless light in plasmonic waveguides. *Phys. Rev. Lett.* **112**, 167401 (2014). 24815668 10.1103/PhysRevLett.112.167401

[38] Hinney, J. et al. Efficient excitation and control of integrated photonic circuits with virtual critical coupling. *Nat. Commun.* **15**, 2741 (2024). 38548757 10.1038/s41467-024-46908-2 PMC10978855

[39] Ra’di, Y., Krasnok, A. & Alù, A. Virtual critical coupling. *ACS Photonics* **7**, 1468–1475 (2020).

[40] Gu, Z. M. et al. Transient non-Hermitian skin effect. *Nat. Commun.* **13**, 7668 (2022). 36509774 10.1038/s41467-022-35448-2 PMC9744917

[41] Smith, D. R. et al. Determination of effective permittivity and permeability of metamaterials from reflection and transmission coefficients. *Phys. Rev. B* **65**, 195104 (2002).

[42] Siegman, A. E. *Lasers*. (Mill Valley: University Science Books, 1986), 111.

[43] Landau, L. & Lifshitz, E. *Electrodynamics of Continuous Media*. 2nd edn. (Amsterdam: Elsevier, 1984).

[44] Luo, L. Y. et al. Non-Hermitian effective medium theory and complex Dirac-like cones. *Opt. Express* **29**, 14345–14353 (2021). 33985157 10.1364/OE.425862

[45] McCall, M. W., Lakhtakia, A. & Weiglhofer, W. S. The negative index of refraction demystified. *Eur. J. Phys.* **23**, 353–359 (2002).

[46] Mocella, V. et al. Self-collimation of light over millimeter-scale distance in a quasi-zero-average-index metamaterial. *Phys. Rev. Lett.* **102**, 133902 (2009). 19392354 10.1103/PhysRevLett.102.133902

[47] Linden, S. et al. Magnetic response of metamaterials at 100 terahertz. *Science* **306**, 1351–1353 (2004). 15550664 10.1126/science.1105371

[48] Zhou, J. et al. Saturation of the magnetic response of split-ring resonators at optical frequencies. *Phys. Rev. Lett.* **95**, 223902 (2005). 16384220 10.1103/PhysRevLett.95.223902

[49] Kuznetsov, A. I. et al. Magnetic light. *Sci. Rep.* **2**, 492 (2012). 22768382 10.1038/srep00492 PMC3389365

[50] Evlyukhin, A. B. et al. Optical response features of Si-nanoparticle arrays. *Phys. Rev. B* **82**, 045404 (2010).

[51] Staude, I. et al. Tailoring directional scattering through magnetic and electric resonances in subwavelength silicon nanodisks. *ACS Nano* **7**, 7824–7832 (2013). 23952969 10.1021/nn402736f

[52] Shen, L. F. & He, S. L. Studies of imaging characteristics for a slab of a lossy left-handed material. *Phys. Lett. A* **309**, 298–305 (2003).

[53] Pendry, J. B., Luo, Y. & Zhao, R. K. Transforming the optical landscape. *Science* **348**, 521–524 (2015). 25931549 10.1126/science.1261244

[54] Ruan, Z. C. & Fan, S. H. Design of subwavelength superscattering nanospheres. *Appl. Phys. Lett.* **98**, 043101 (2011).

[55] Smith, D. R., Pendry, J. B. & Wiltshire, M. C. K. Metamaterials and negative refractive index. *Science* **305**, 788–792 (2004). 15297655 10.1126/science.1096796

[56] Enkrich, C. et al. Magnetic metamaterials at telecommunication and visible frequencies. *Phys. Rev. Lett.* **95**, 203901 (2005). 16384056 10.1103/PhysRevLett.95.203901

[57] Dolling, G. et al. Low-loss negative-index metamaterial at telecommunication wavelengths. *Opt. Lett.* **31**, 1800–1802 (2006). 16729075 10.1364/ol.31.001800

[58] Leonhardt, U. Optical conformal mapping. *Science* **312**, 1777–1780 (2006). 16728596 10.1126/science.1126493

[59] Sweeney, W. R. et al. Perfectly absorbing exceptional points and chiral absorbers. *Phys. Rev. Lett.* **122**, 093901 (2019). 30932516 10.1103/PhysRevLett.122.093901

[60] Hughes, T. W. et al. Forward-mode differentiation of Maxwell’s equations. *ACS Photonics* **6**, 3010–3016 (2019).

[61] Rumpf, R. C. Simple implementation of arbitrarily shaped total-field/scattered-field regions in finite-difference frequency-domain. *Prog. Electromagn. Res. B* **36**, 221–248 (2012).

[62] Whittaker, D. M. & Culshaw, I. S. Scattering-matrix treatment of patterned multilayer photonic structures. *Phys. Rev. B* **60**, 2610–2618 (1999).

