# Supplementary material for: Ideal optical antimatter using passive lossy materials under complex frequency excitation
Source: Light Sci Appl. 2026 Jan 4;15:48. doi: 10.1038/s41377-025-02137-w (PMC12764775; doi:10.1038/s41377-025-02137-w)
Supplement: Supplementary file 1 — Supplemental Material [file 41377_2025_2137_MOESM1_ESM.pdf]

Supplementary Material for: "Ideal optical antimatter using passive lossy materials under complex frequency excitation"

Olivia Y. Long,[1, 2, *] Peter B. Catrysse,[2] Seunghoon Han,[3, 4] and Shanhui Fan[1, 2, 5, †]

[1] *Department of Applied Physics, Stanford University, Stanford, California 94305, USA*
[2] *Edward L. Ginzton Laboratory, Stanford University, Stanford, California 94305, USA*
[3] *Samsung Advanced Institute of Technology, Samsung Electronics, Suwon-si 16678, South Korea*
[4] *Semiconductor R&D Center, Samsung Electronics, Hwaseong-si 18448, South Korea*
[5] *Department of Electrical Engineering, Stanford University, Stanford, California 94305, USA*

(Dated: November 6, 2025)

CONTENTS

I. Derivation of matrix expression in main text 1

II. Derivation of α interval over which $\mathbf{x} > 0$ 2

III. Calculation of null-space vector 2

IV. Electromagnetic field demonstrations for different geometries at complex frequency 3

V. Total-Field/Scattered-Field simulation details 4

VI. Virtual gain in Lorentz-Drude material at complex frequency 5

VII. Lensing using Imperfect Complementary Media 5

VIII. Perfect lens using source with finite bandwidth 8

9

References 9

I. DERIVATION OF MATRIX EXPRESSION IN MAIN TEXT

In this section, we derive the matrix M and vector $\mathbf{b}$ given in Eq. 10 of the main text. We start with $\epsilon(\omega) = C + iD$ where $\omega = \omega' + i\omega''$:

$$\epsilon(\omega) = 1 + \frac{\omega_p^2}{A - iB} = C + iD \tag{S1}$$

Here, we have used $A \equiv -[\omega_0^2 - \omega'^2 + \omega''(\omega'' + \gamma)]$ and $B \equiv \omega'(2\omega'' + \gamma)$. Multiplying both sides of Eq. S1 by $A - iB$, we have:

$$A - iB + \omega_p^2 = (C + iD)(A - iB) \tag{S2}$$

Matching the real and imaginary parts, we get:

$$A + \omega_p^2 = AC + BD \tag{S3}$$
$$-B = AD - BC \tag{S4}$$

* olong@stanford.edu
† shanhui@stanford.edu

Expanding out the expressions for A and B, the two equations become:

$$[\omega'^2 - \omega_0^2 - \omega''(\omega'' + \gamma)] + \omega_p^2 = C[\omega'^2 - \omega_0^2 - \omega''(\omega'')] + \omega'(2\omega'' + \gamma)D \tag{S5}$$

$$-\omega'(2\omega'' + \gamma) = D[\omega'^2 - \omega_0^2 - \omega''(\omega'' + \gamma)] - \omega'(2\omega'' + \gamma)C \tag{S6}$$

Combining like terms and writing in matrix form, we arrive at the matrix expression in Eq. 10–13 of the main text:

$$\begin{bmatrix} C-1 & 1 & \omega''(C-1) - \omega' D \\ D & 0 & \omega'(C-1) + \omega'' D \end{bmatrix} \begin{bmatrix} \omega_0^2 \\ \omega_p^2 \\ \gamma \end{bmatrix} = \begin{bmatrix} (C-1)(\omega'^2 - \omega''^2) + 2\omega'\omega'' D \\ D(\omega'^2 - \omega''^2) - 2\omega'\omega''(C-1) \end{bmatrix} \tag{S7}$$

II. DERIVATION OF α INTERVAL OVER WHICH $\mathbf{x} > 0$

In this section, we derive the range of α in which the components of $\mathbf{x}$ are all positive, where α is the free variable from Eq. 15 of the main text. The derivation here provides additional information beyond Theorem 1 in the main text, which states that:

$$\exists\, \alpha : \quad x_k(\alpha) > 0 \quad \forall\, k. \tag{S8}$$

Note that the component $(x_n)_2 > 0$ since $\omega' > 0$. Thus, for all $\alpha > 0$, $x_2(\alpha) > 0$ since $(x_p)_2 = 0$.

For $k \neq 2$, $(x_n)_k$ may be > 0 or < 0 depending on the values of C and D. If $(x_n)_k > 0$, $x_k(\alpha) > 0$ when:

$$\alpha > -\frac{(x_p)_k}{(x_n)_k} \tag{S9}$$

If $(x_n)_k < 0$, $x_k(\alpha) > 0$ when:

$$\alpha < -\frac{(x_p)_k}{(x_n)_k} \tag{S10}$$

Hence, the values of α satisfying Eq. (S8) lie in the interval:

$$\alpha \in \left(\max_{k \in I^+} \left(-\tfrac{(x_p)_k}{(x_n)_k} \right), \min_{k \in I^-} \left(-\tfrac{(x_p)_k}{(x_n)_k} \right) \right) \tag{S11}$$

where $I^+ = \{\, k \in \{1,3\} \mid (x_n)_k > 0\}$ and $I^- = \{\, k \in \{1,3\} \mid (x_n)_k < 0\}$.

Since $(x_p)_k > 0$ for $k \neq 2$, the interval in Eq. (S11) has a negative lower bound and a positive upper bound, which guarantees that it is non-empty. Thus, there exists $\alpha > 0$ in the interval of Eq. (S11), satisfying $x_2(\alpha) > 0$ and completing the proof of Eq. (S8). In other words, we have shown that Eqns. 10–13 in the main text can be satisfied by a passive, lossy material for arbitrary $C, D \in \mathbb{R}$.

III. CALCULATION OF NULL-SPACE VECTOR

In this section, we calculate the cross product of the vectors $\mathbf{r}_1, \mathbf{r}_2$ to find a nullspace vector $\mathbf{x}_n$ (Eq. 16 of main text):

$$\begin{aligned} \mathbf{x}_n = \mathbf{r}_1 \times \mathbf{r}_2 &= \begin{vmatrix} \mathbf{i} & \mathbf{j} & \mathbf{k} \\ C-1 & 1 & \omega''(C-1) - \omega' D \\ D & 0 & \omega'(C-1) + \omega'' D \end{vmatrix} \\ &= [\omega'(C-1) + \omega'' D]\mathbf{i} - \omega'[D^2 + (C-1)^2]\mathbf{j} - D\mathbf{k}. \end{aligned} \tag{S12}$$

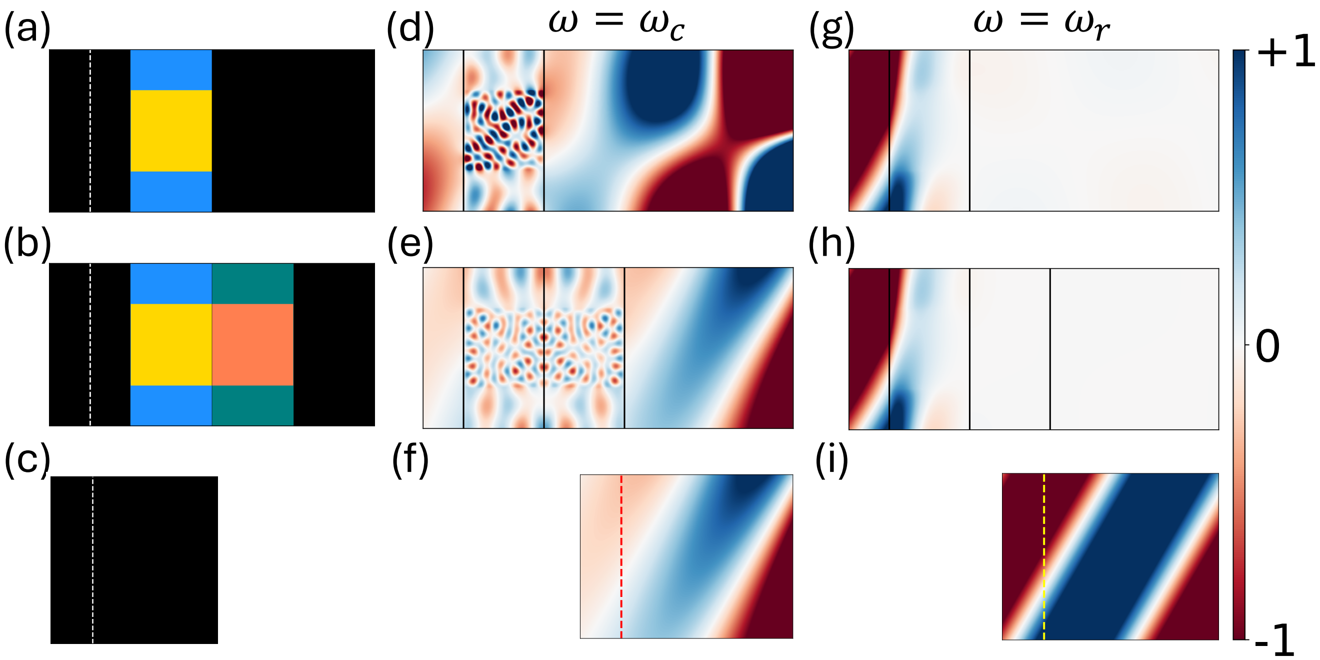

FIG. S1. Optical antimatter for planar inhomogeneity along the transverse direction. In panels (a)–(c), the colors blue, teal, yellow and orange correspond to Media 1, 2, 3, 4, as described in Table I of the main text, respectively. Black denotes vacuum. (a) Schematic of structure with a layer of Medium 3 (yellow) with thickness 0.6 μm along the transverse direction is embedded in Medium 1 (blue), situated in a background of air. The slab has thickness 0.6 μm along the propagation direction. Dashed white line indicates location of TM-polarized plane wave source. (b) Schematic of structure placed adjacent to its complementary counterpart. (c) Free space configuration with plane wave source. (d)–(f) TM-polarized Re[H_z] field patterns at the complex frequency $\omega = \omega_c$ for the configurations in (a), (b), and (c), respectively. (g)–(i) TM-polarized Re[H_z] field patterns at the real frequency $\omega = \omega_r$ for the configurations in (a), (b), and (c), respectively. In (f) and (i), the red and yellow dashed lines mark the effective location of the optically cancelled complementary media pair, respectively. For each ω, field plots are normalized to the same maximum and minimum values. All field plots were generated using FDFD [40], with Bloch boundary conditions in the transverse (y) direction and PML boundary conditions in the propagation (x) direction. Plots show 0th diffraction order in free space.

IV. ELECTROMAGNETIC FIELD DEMONSTRATIONS FOR DIFFERENT GEOMETRIES AT COMPLEX FREQUENCY

In Fig. S1, we demonstrate optical antimatter using another structure comprised of a slab with planar inhomogeneity along the transverse direction. In panel (a), a layer of Medium 3 (yellow) with thickness 0.6 μm along the transverse direction is embedded in Medium 1 (blue), situated in a background of air. The slab has thickness 0.6 μm along the propagation direction. The top right panel shows that the fields transmitted through the structure strongly deviate from the incident plane wave. By placing the complementary slab structure adjacent to the single slab, we are once again able to add optical antimatter to optically cancel the scattering from the first slab. As shown in panel (e), we see that the transmitted fields emerging to the right of the complementary pair match the free-space case in Fig. S1(f).

In Fig. S2(a)–(b), we demonstrate the field pattern recovery of a scatterer placed adjacent to a pair of complementary media. In the top panel of (a), we show the schematic of the setup. The top panel of (b) shows the resulting field pattern at complex frequency $\omega = \omega_c$. We can compare the field pattern to that of just the scatterer shown schematically in the lower panel of (a), and we see that the emerging fields on the right hand side are the same [lower panel of (b)]. In Figs. S2(c)–(d), we demonstrate the optical antimatter of two nested sets of complementary media, which cancel pairwise, as imagined in the original proposal [1]. The configuration is shown schematically in the top panel of (c). The inner two slabs form a complementary pair and effectively cancel each other, while the outer two slabs cancel. As a result, the fields on the right hand side of the structure emerge as if unperturbed, as seen in the top panel of (d). We can compare the fields to those of the incident plane wave propagating in free-space, shown schematically in the lower panel of (c), and we see that the fields on the right side of the dashed red line in the lower

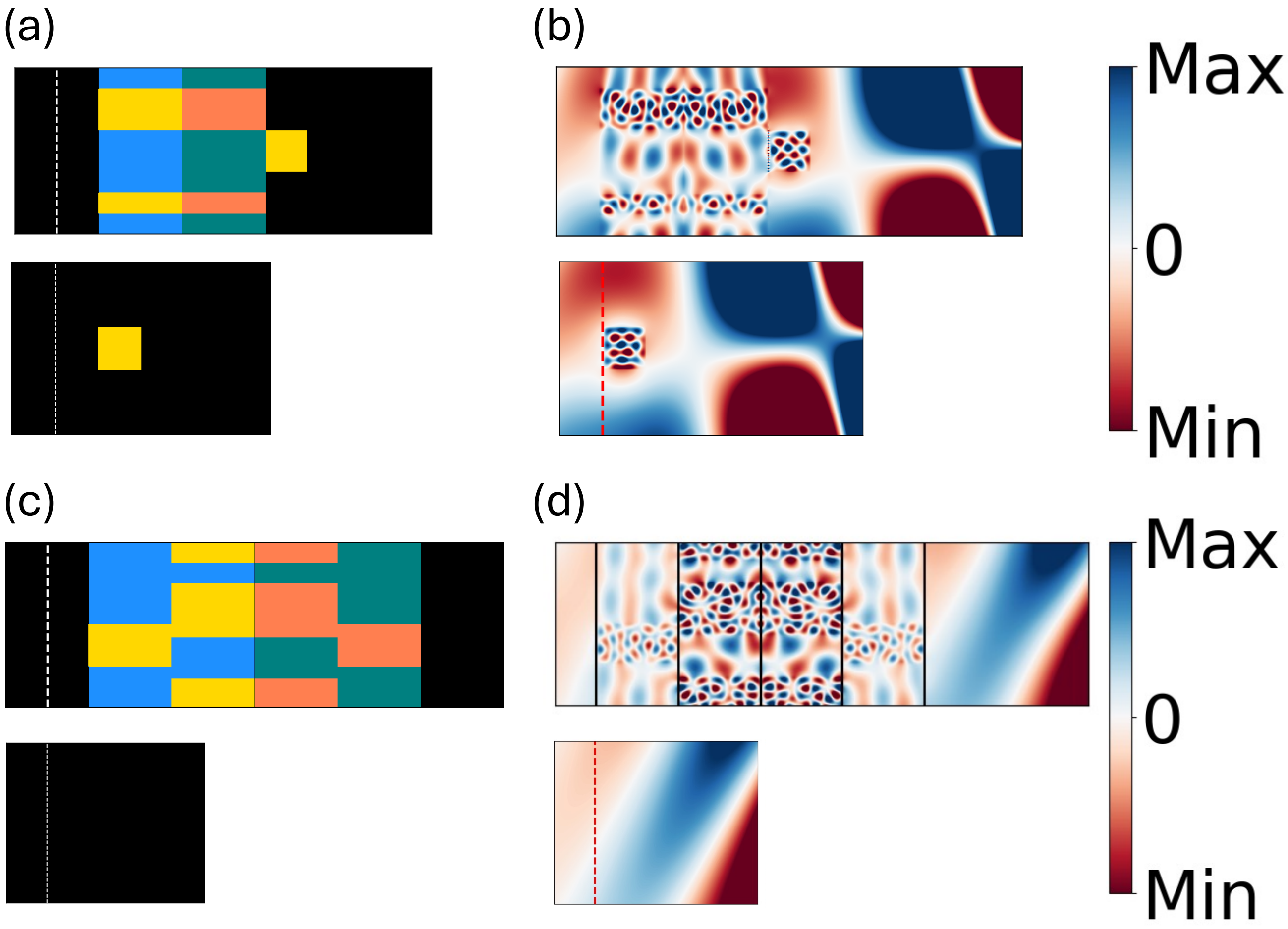

FIG. S2. Field pattern recovery of a scatterer using optical antimatter. (a) Schematic of complementary pair placed in front of scatterer in free space (top panel) and equivalent configuration of the scatterer in free space, with the space occupied by the complementary pair removed (bottom panel). (b) TM-polarized Re$[H_z]$ field patterns at the complex frequency $\omega = \omega_c$ for the configurations shown in (a). (c) Nested configuration of two pairs of complementary media (top panel) and equivalent configuration in free space (bottom panel). The two innermost layers and the two outermost layers for complementary pair. Taken pairwise, the overall scattering pattern is cancelled. (d) TM-polarized Re$[H_z]$ field patterns at the complex frequency $\omega = \omega_c$ for the configurations shown in (c). In all panels showing schematics, the dotted white line indicates plane wave source location. The dotted red lines in the field patterns indicate effective location of the optical antimatter structure that is rendered invisible.

panel of (d) match those on the right side of the slab structure [top panel of (d)].

V. TOTAL-FIELD/SCATTERED-FIELD SIMULATION DETAILS

Fig. S3 shows the setup for computing the scattering cross section using the TFSF framework within our FDFD simulations. The scattering cross sections of the structures shown in Fig. 3 of the main text were computed by taking the line integral of the Poynting vector over the bounding box (depicted in red) outside the TFSF box and normalizing by the incident power S_x inside box with no scatterer. The TFSF code was modified to accommodate for background media with arbitrary permittivity and permeability values.

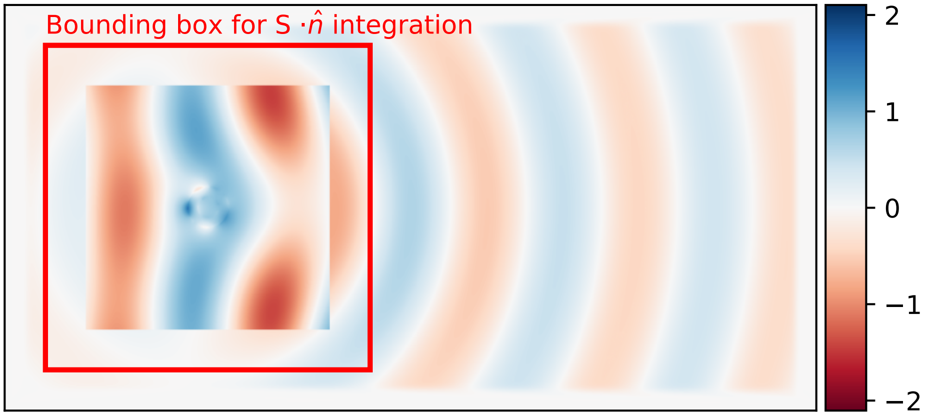

FIG. S3. Schematic of Total Field Scattered Field setup in FDFD simulations. The outgoing Poynting flux is integrated along the bounding box shown in red.

VI. VIRTUAL GAIN IN LORENTZ-DRUDE MATERIAL AT COMPLEX FREQUENCY

In this section, we derive the conditions for achieving $\text{Im}[\epsilon] < 0$ (virtual gain) in a Lorentz-Drude material, without using intrinsic material gain. At a given complex frequency $\omega = \omega' + i\omega''$, we have:

$$\epsilon(\omega) = 1 + \frac{\omega_p^2}{\omega_0^2 - \omega^2 - i\omega\gamma} = 1 + \frac{\omega_p^2}{(\omega_0^2 - \omega'^2 + \omega''^2 + \omega''\gamma) - i\omega'(2\omega'' + \gamma)} \tag{S13}$$

$$= 1 + \frac{\omega_p^2}{A - iB} = \frac{[A(A + \omega_p^2) + B^2] + iB\omega_p^2}{A^2 + B^2} \tag{S14}$$

where A, B are defined in Section I above. To achieve $\text{Im}[\epsilon] < 0$, the condition is:

$$B\omega_p^2 < 0 \tag{S15}$$

$$\omega_p^2\omega'(2\omega'' + \gamma) < 0 \tag{S16}$$

Since we would like to have a material with no intrinsic gain (i.e. $\omega_p^2 > 0$), the condition becomes:

$$\omega'(2\omega'' + \gamma) < 0 \tag{S17}$$

Since $\omega' > 0$, this gives us the condition on γ:

$$\gamma < -2\omega'' \tag{S18}$$

Thus, for $\gamma > 0$ as must be true in a physical Lorentz-Drude material, we must have $\omega'' < 0$, which corresponds to a temporally decaying wave. This is also the reason we choose to work with decaying waves rather than growing waves in time to achieve complementary media and optical antimatter effects.

VII. LENSING USING IMPERFECT COMPLEMENTARY MEDIA

In this section, we study the performance of the perfect lens structure of Fig. 2(a) in the main text using imperfect complementary media.

In Figs. S4 and S5, we perturb the real and imaginary parts of μ_2 in Medium 2 (central slab), respectively. We first perturb $\text{Re}[\mu_2]$ in Fig. S4. Depending on the direction of perturbation (i.e. increasing or decreasing the magnitude of $\text{Re}[\mu_2]$), the wavevector in Medium 2 can exhibit either effective gain or loss, respectively. This is shown in Fig. S4(a), where the magnitude of $\text{Re}[\mu_2]$ is decreased to 80% of the ideal value, yielding effective gain in the system [see transmission plot in Fig. S4(d)]. In Figs. S4(b) and (c), the magnitude of $\text{Re}[\mu_2]$ is increased to 110% and 150% of the ideal value, respectively. In both cases, we see that the system now exhibits effective loss as shown in the transmission plots of Figs. S4(e) and (f).

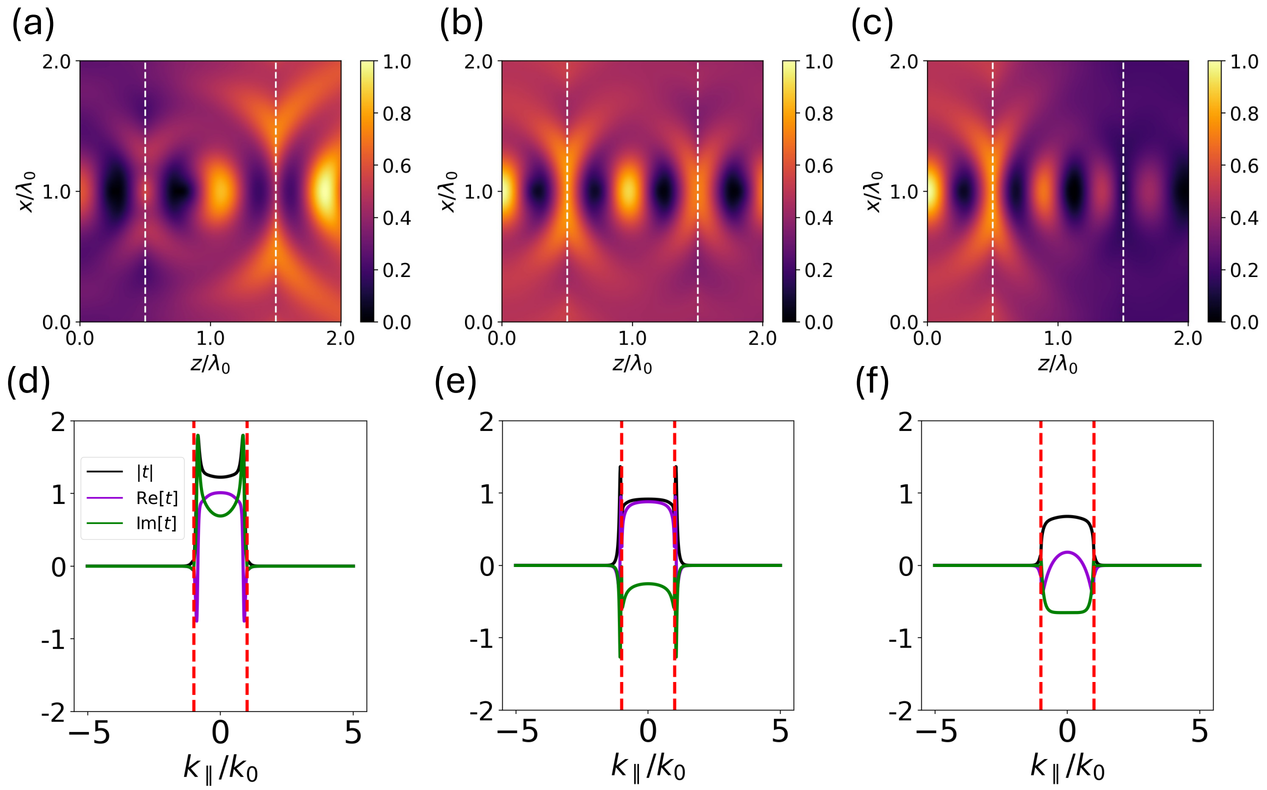

FIG. S4. Performance of perfect lens structure shown in Fig. 2(a) of main text using imperfect complementary media where Re[μ_2] is perturbed from the ideal value. Magnitude of Poynting vector for (a) $\mu_2 = -0.8(6) - 1.911i$ (b) $\mu_2 = -1.1(6) - 1.911i$ and (c) $\mu_2 = -1.5(6) - 1.911i$ with only propagating waves plotted for visual clarity. (d)–(f) Transmission coefficient as function of parallel wavevector $k_\parallel$ through corresponding structure.

However, as shown in the transmission plots of Figs. S4 (d)–(f), the transmission of the evanescent waves are greatly reduced. The high sensitivity of the evanescent waves to effective losses has been studied in Ref. [3], and is the reason why it has historically been so difficult to observe perfect lensing experimentally.

We now perturb Im[μ_2] in Fig. S5. From Figs. S5 (a)–(c), we see that as Im[μ_2] is varied from 20% of its ideal value to 80%, the refocusing phenomenon is gradually recovered for propagating waves. Again, due to the complex wavevector in Medium 2, the transmission of the evanescent waves is reduced as in the case of Fig. S4. Similar trends were also observed for perturbations in Re[ϵ_2] and Im[ϵ_2].

However, even with imperfect complementary media, a portion of evanescent waves can still be recovered if the effective negative index material is made thin enough. To demonstrate this, in Fig. S6, we perturb Re[ϵ_2] by 10% of its ideal value. By using progressively thinner slabs, we observe that subwavelength imaging can still be achieved, since more evanescent wave components can be recovered. In Fig. S6(c), we see that when the central slab has thickness $d_1 = 0.1\lambda_0$, much of the evanescent waves are transmitted. Similar results are obtained for the analogous perturbation to the permeability μ_2.

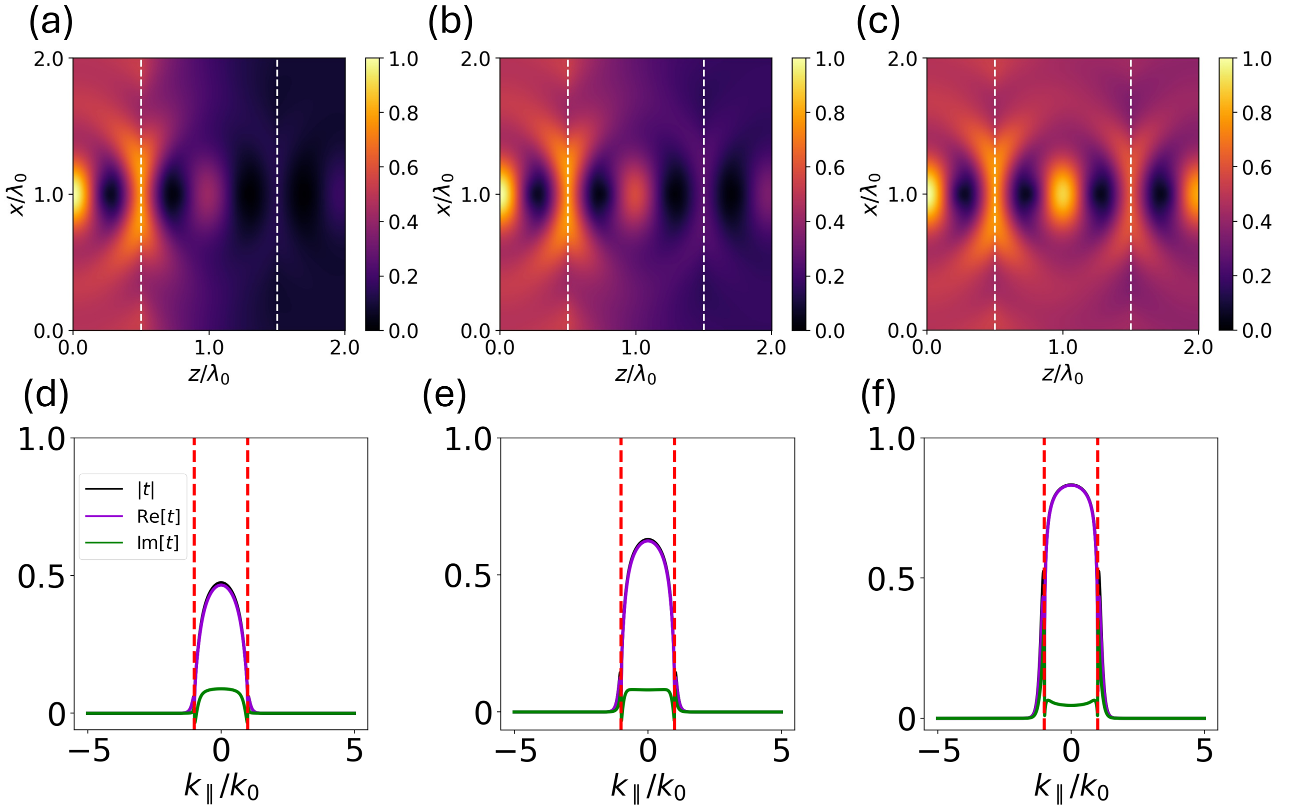

FIG. S5. Performance of perfect lens structure shown in Fig. 2(a) of main text using imperfect complementary media where $\mathrm{Im}[\mu_2]$ is perturbed from the ideal value. Magnitude of Poynting vector for (a) $\mu_2 = -6 - 0.2(1.911)i$ (b) $\mu_2 = -6 - 0.5(1.911)i$ and (c) $\mu_2 = -6 - 0.8(1.911)i$ with only propagating waves plotted for visual clarity. (d)–(f) Transmission coefficient as function of parallel wavevector $k_\parallel$ through corresponding structure.

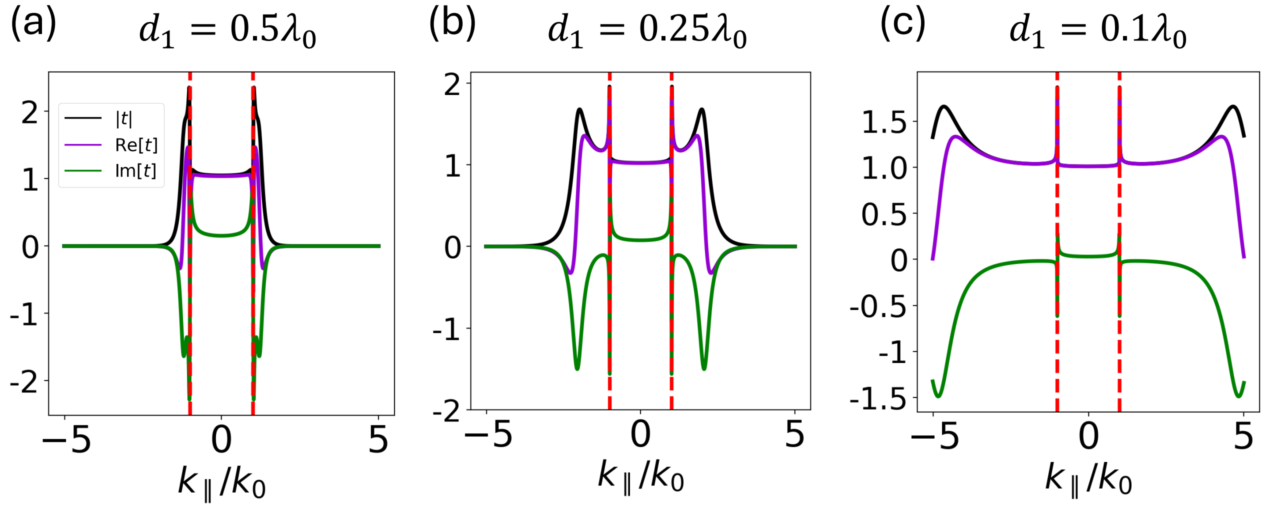

FIG. S6. Lens using imperfect complementary media and slabs with different thicknesses for d_1, as defined in Fig. 2 of the main text (central slab). For each case, $d_0 = 0.5d_1 = d_2$. The first and third layers consist of Medium 1, while the second layer is now an imperfect complementary medium, with parameters $\epsilon_2 = -3.6 - 1.274i, \mu_2 = -6 - 1.911i$, corresponding to a 10% perturbation of $\mathrm{Re}[\epsilon_2]$ from its ideal value. (a) $d_1 = 0.5\lambda_0$ (b) $d_1 = 0.25\lambda_0$ (c) $d_1 = 0.1\lambda_0$.

VIII. PERFECT LENS USING SOURCE WITH FINITE BANDWIDTH

In this section, we provide simulations of perfect lens performance for a Gaussian source with a finite bandwidth. We use the Lorentz-Drude material parameters presented in Table I of the main text for the layers of Medium 1 and Medium 2 (structure shown in Fig. 2(a) of main text), which will give different values of $\epsilon_1, \mu_1, \epsilon_2, \mu_2$ as ω is varied due to the dispersion. We assume the bandwidths are Gaussian in the frequency domain and characterize them using their full-width half-max (FWHM). The FWHM values we plot are: $10^{-2}\omega_0, 10^{-3}\omega_0, 10^{-4}\omega_0, 10^{-6}\omega_0$ where $\omega_0 = \text{Re}[\omega_c]$ as defined in the main text, corresponding to a central wavelength of $\lambda_0 = 1550$ nm. Using the FWHM value, we define the Gaussian in the frequency domain as:

$$S(\omega) = e^{-(\omega-\omega_c)^2/2\sigma_\omega^2} \tag{S19}$$

where $\sigma_\omega \equiv \text{FWHM}/2\sqrt{2\ln(2)}$. To simulate the transmission for a finite bandwidth source, we first compute the transmission coefficient $t(\omega, k_\parallel)$ for $\omega \in [\omega_c - 2.5\sigma_\omega, \omega_c + 2.5\sigma_\omega]$ with step size $\Delta\omega$. Then, we multiply the result for each ω by the corresponding weight $S(\omega)\Delta\omega$ and normalize by $\sum_\omega S(\omega)\Delta\omega$. We note that only $\text{Re}[\omega]$ is varied and the decay rate is held constant ($\text{Im}[\omega] = -4 \times 10^{14}$).

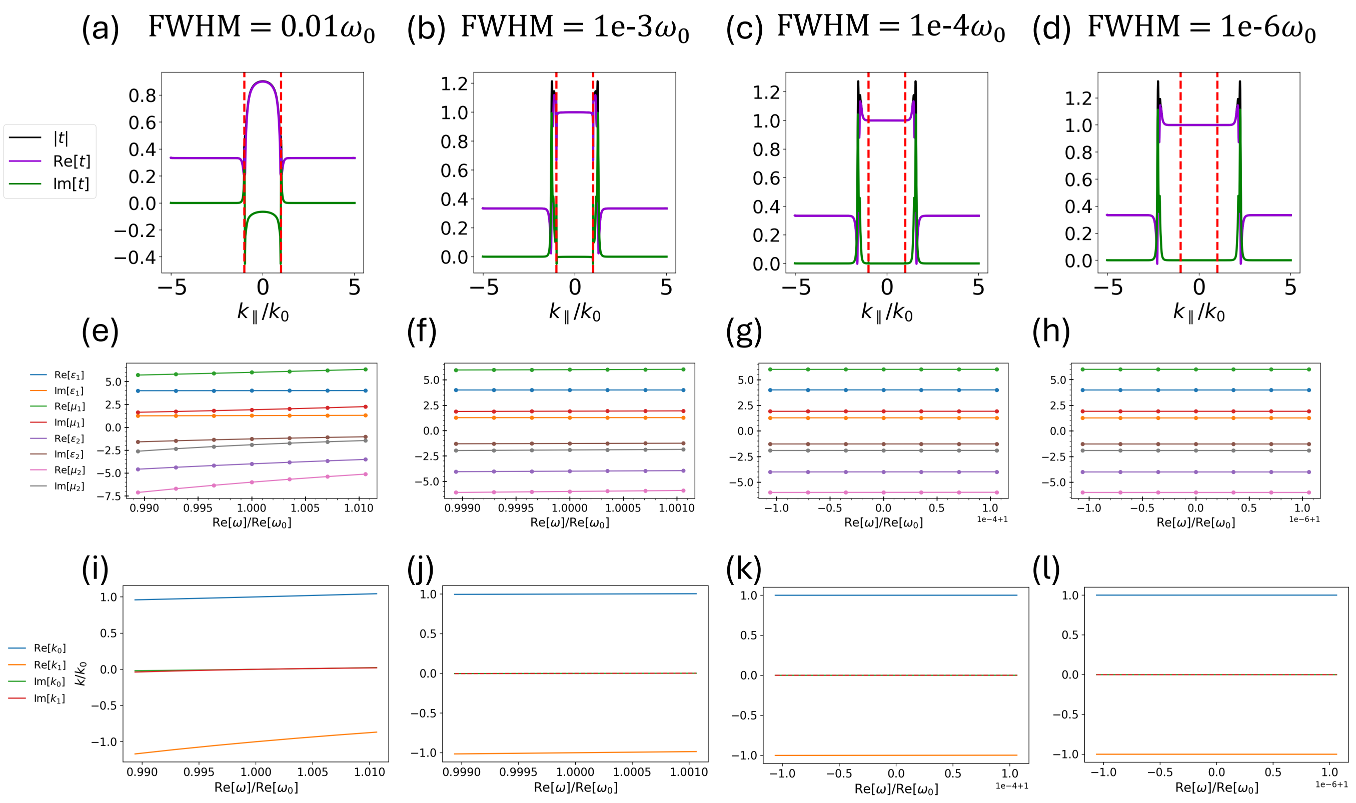

FIG. S7. Lensing with structure shown in Fig. 2(a) of main text using finite bandwidth source. Transmission coefficient through structure for sources with FWHM values of (a) $0.01\omega_0$ (b) 1e-3ω_0 (c) 1e-4ω_0 (d) 1e-6ω_0. (e)–(h) Real and imaginary components of $\epsilon_1, \mu_1, \epsilon_2, \mu_2$ as function of frequency for corresponding frequency bandwidths. (i)–(l) Real and imaginary components of k_0 and k_1 as function of frequency for corresponding frequency bandwidths.

In Figs. S7(a)–(d), we plot the transmission coefficients as a function of the parallel wavevector $k_\parallel$ for the different bandwidths. As the bandwidth becomes narrower, the transmission approaches that of a perfect lens since the $k_\parallel$ range over which $|t| \approx 1$ becomes larger and begins to include evanescent waves starting from FWHM = 1e-4ω_0. This is because as the bandwidth gets narrower, the values of $\epsilon_1, \mu_1, \epsilon_2, \mu_2$ approach the ideal values that will give perfect lensing at the given ω_c. For wider bandwidths, the material dispersion causes the $\epsilon_1, \mu_1, \epsilon_2, \mu_2$ to differ from the ideal values, resulting in lossy wavevectors in both media i.e. $\text{Im}[k_0], \text{Im}[k_1] \neq 0$. Due to the presence of effective loss, the evanescent waves cannot be perfectly recovered, as has been shown in Section VII as well. However, partial recovery of the evanescent waves is still possible, since $|t(k_\parallel)| \neq 0$ for $k_\parallel > k_0$, as shown in the plots.

In Figs. S7 (e)–(h), we show the material dispersion effects on the values of the real and imaginary parts of $\epsilon_1, \mu_1, \epsilon_2, \mu_2$. We see that for the widest bandwidth (FWHM = $0.01\omega_0$), the dispersion effects are most pronounced. For example, in Fig. S7(e), we see that the values of $\mathrm{Re}[\mu_2], \mathrm{Re}[\epsilon_2], \mathrm{Im}[\mu_1]$ are not constant across the entire bandwidth and have noticeable slopes. These differences from the ideal case (which corresponds to $\mathrm{Re}[\omega]/\mathrm{Re}[\omega_0] = 1$ on the x-axis in the plots), cause the wavevectors in both media to become complex instead of lossless. As a consequence, the lensing is no longer perfect lensing. To show this, The real and imaginary parts of k_0, k_1 are plotted as a function of frequency in Figs. S7 (i)–(l) for the corresponding frequency bandwidths.

In typical experimental setups, the bandwidth for a 1550 nm source is $\approx$ 10 MHz, corresponding to FWHM $\approx 5 \times 10^{-8}\omega_0$ [4]. Thus, such setups with narrower bandwidths would demonstrate behavior closer to the perfect lens than shown in the simulations.

Supplementary information accompanies the manuscript on the Light: Science & Applications website (`http://www.nature.com/lsa`)

REFERENCES

[1] J. B. Pendry and S. A. Ramakrishna, "Focusing light using negative refraction," *Journal of Physics: Condensed Matter* **15**, 6345 (2003).

[2] T. W. Hughes, I. A. Williamson, M. Minkov, and S. Fan, "Forward-mode differentiation of Maxwell's equations," *ACS Photonics* **6**, 3010–3016 (2019).

[3] L. Shen and S. He, "Studies of imaging characteristics for a slab of a lossy left-handed material," *Physics Letters A* **309**, 298 (2003).

[4] Thorlabs, "Pigtailed, Distributed Feedback (DFB) Single-Frequency Lasers with Internal Isolator," *Thorlabs Product Page*, `https://www.thorlabs.com/newgrouppage9.cfm?objectgroup_id=7928`, accessed Aug. 31, 2025.
